# Nanotechnology-Assisted Immunogenic Cell Death for Effective Cancer Immunotherapy

**DOI:** 10.3390/vaccines11091440

**Published:** 2023-08-31

**Authors:** Yichen Guo, Rong Ma, Mengzhe Zhang, Yongjian Cao, Zhenzhong Zhang, Weijing Yang

**Affiliations:** 1 School of Pharmaceutical Sciences, Zhengzhou University, Zhengzhou 450001, China; gyc0222@gs.zzu.edu.cn (Y.G.); mr4721@gs.zzu.edu.cn (R.M.); zmz15670910876@gs.zzu.edu.cn (M.Z.); cyong@gs.zzu.edu.cn (Y.C.); 2 Key Laboratory of Targeting Therapy and Diagnosis for Critical Diseases, Zhengzhou 450001, China

**Keywords:** immunogenic cell death, nanotechnology, tumor vaccines, cancer immunotherapy

## Abstract

Tumor vaccines have been used to treat cancer. How to efficiently induce tumor-associated antigens (TAAs) secretion with host immune system activation is a key issue in achieving high antitumor immunity. Immunogenic cell death (ICD) is a process in which tumor cells upon an external stimulus change from non-immunogenic to immunogenic, leading to enhanced antitumor immune responses. The immune properties of ICD are damage-associated molecular patterns and TAA secretion, which can further promote dendritic cell maturation and antigen presentation to T cells for adaptive immune response provocation. In this review, we mainly summarize the latest studies focusing on nanotechnology-mediated ICD for effective cancer immunotherapy as well as point out the challenges.

## 1. Introduction

Cancer immunotherapy is emerging as a promising treatment strategy for many cancer types [1,2,3]. It mainly depends on activating host immune response to kill tumor cells, which is in contrast to traditional therapeutic approaches. Immunotherapy approaches include immunogenic cell death (ICD) inducement, cancer vaccination, and adoptive T cell therapy [4,5,6]. ICD is used to elicit host immune responses as a result of dying tumor cells becoming immunogenic due to external death-promoting stimuli [7]. During this process, damage-associated molecular patterns (DAMPs) and tumor-associated antigens (TAAs) are released and subsequently captured by macrophages and dendritic cells (DCs) [8,9]. After processing, the antigen is presented to adaptive immune cells to activate host-specific immune response. DAMPs include adenosine triphosphate (ATP) as the “find me” signal, calreticulin (CRT) as the “eat me” signal, and high mobility group box 1 (HMGB1) for DC activation. In addition, cytokines (e.g., tumor necrosis factor-α (TNF-α) and interleukin-6 (IL-6)) are released during tumor ICD accompanied by a strong inflammatory response [10]. Nowadays, a series of tumor therapies, such as radiotherapy (RT), photodynamic therapy (PDT), hyperthermia therapy (HT), chemodynamic therapy (CDT), and chemotherapy, can be used in ICD-mediated cancer immunotherapy.

**Figure 1:**
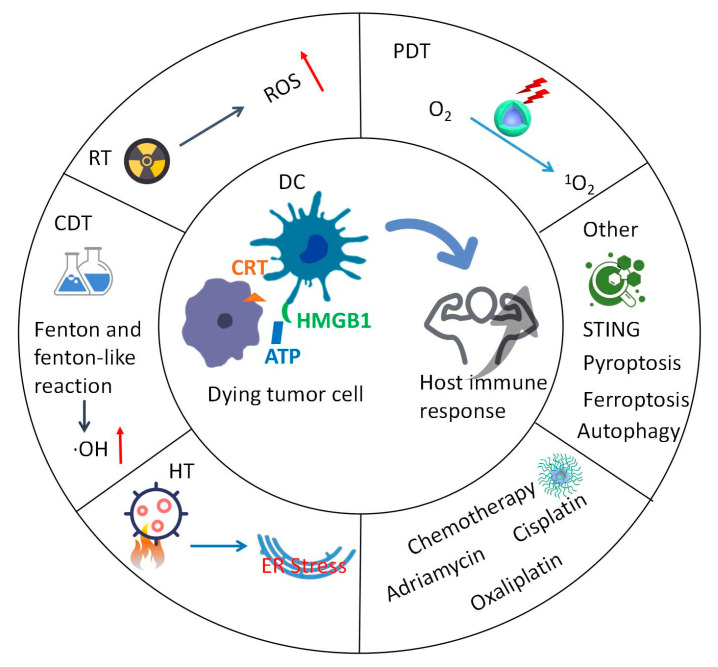
ICD can be induced by a series of treatment modalities, including RT, PDT, HT, CDT, and chemotherapy. RT kills tumor cell by direct DNA or indirect ROS damage. PDT is induced by a cascade of ROS reactions. HT means heating tissues to a relatively high temperature with ER stress. The dying tumor cells expose CRT and release DAMPs (e.g., ATP and HMGB1) which can recruit and activate DC to enhance host immune response. ICD: immunogenic cell death; RT: radiotherapy; PDT: photodynamic therapy; HT: hyperthermia therapy; CDT: chemodynamic therapy; DAMPs: damage-associated molecular patterns; DC: dendritic cell; ROS: reactive oxygen species; O_2_: oxygen; ^1^O_2_: singlet oxygen; CRT: Calreticulin; HMGB1: High Mobility Group Box 1; ATP: adenosine triphosphate; •OH: hydroxyl radical; STING: Cyclic GMP-AMP (cGAS)-stimulator of interferon genes.

Nanomedicines have long circulation time, good biocompatibility, and low side effects in vivo compared with free drugs, thus generally being exploited in antitumor treatment [11,12]. In addition, nanoparticles can also accumulate into tumors through the enhanced permeability and retention effect [13,14,15]. Surface modification of nanoparticles is able to increase their cellular internalization, which is a prerequisite for triggering ICD [16,17]. Moreover, drug loading content can also be improved via electrostatic interaction between nanoparticles and drugs with controlled release when the responsive groups encounter an internal or external stimulus. Thus, nanomedicines have huge advantages in drug delivery with robust antitumor efficacy during cancer immunotherapy [18,19,20]. The general nanocarriers include polymeric/inorganic nanoparticles, biomimetic nanosystems, nanogels, covalent organic frameworks, nanocomplexes, etc. [21,22,23,24,25]. Herein, we mainly discuss the nanotechnology-assisted therapeutic strategies for tumor ICD based on RT, PDT, HT, CDT, and chemotherapy (Figure 1).

## 2. Radiotherapy

Radiotherapy is a powerful therapeutic modality of cancer which can kill tumor cells through direct DNA damage and indirect reactive oxygen species (ROS) damage without tissue penetration limitation [6,26,27,28]. Recently, it has been reported that RT can induce ICD with TAA and DAMP release, thereby triggering DC maturation and eliciting antitumor immune responses [29,30,31,32,33,34,35,36,37,38]. It is widely reported that the coordinated high-order metal elements in nanocrystals can induce tumor ICD via radiotherapy. For example, Huang et al. reported bi-functional coordination nanorod (ZGd-NR) self-assembly from zoledronic acid and Gd^3+^ (gadolinium). ZGd-NRs efficiently deposited X-rays and generated hydroxyl radicals, thereby inducing an effective ICD [28]. In addition, ZGd-NRs depleted tumor-associated macrophages (TAMs) and reprogrammed the immunosuppressive microenvironment. In another report, Deng et al. synthesized manganese dioxide (MnO_2_) nanoparticles loaded with programmed death-ligand 1 antibody (aPD-L1) (αPDL1@MnO_2_) by biomineralization [33]. After αPDL1@MnO_2_ accumulated in the tumor site, it induced ICD under X-ray irradiation. Simultaneously, MnO_2_ catalyzed hydrogen peroxide (H_2_O_2_) to produce oxygen with tumor hypoxia alleviation and radiotherapy efficacy enhancement, and the released Mn^2+^ activated the cyclic GMP-AMP (cGAS)-stimulator of interferon genes (STING) pathway and triggered tumor-specific immunity. The released αPD-L1 blocked the immune checkpoint, resulting in the reinvigoration of exhausted CD8^+^ T cells. αPDL1@MnO_2_ inhibited tumor growth as well as metastasis by reprogramming the immunosuppressive microenvironment. Choi et al. synthesized radiosensitive snowflake-like Au nanocarriers (S-AuNC) with aPD-L1 encapsulation [39]. Under X-ray irradiation, S-AuNC nanoparticles produced ROS, promoted endoplasmic reticulum stress, and induced ICD. The released aPD-L1 revealed a synergistic antitumor efficacy and minimized systemic immune-related adverse effects. Li et al. developed a multifunctional MOF (metal–organic framework) structure (named TZM) using Ta (tantalum) and Zr (zirconium) as metal nodes and tetrakis (4-carboxyphenyl)porphyrin (TCPP) as a photosensitizer ligand [35]. TZM preferentially accumulated at tumor sites, absorbed X-ray energy, and effectively generated hydroxyl radicals. The Ta-Zr co-doping design promoted TZM to transfer energy to the photosensitizer TCPP to sensitize the production of singlet oxygen (^1^O_2_) for radiodynamic therapy (RDT). Further investigation indicated that TZM-mediated radio-/radiodynamic therapy with tumor ICD promoted DC maturation and up-regulated PD-L1 expression through the cGAS-STING pathway with antitumor immune response amplification. In addition, combination with chemoimmunotherapy is a general strategy during radiotherapy. Lu et al. constructed a mesoporous upconversion nanophosphor (UCNP) based on bismuth (Bi) with doxorubicin (DOX) encapsulation for both radiotherapy- and chemotherapy-mediated ICD as well as TAM polarization [40]. In vivo studies indicated that the nanoplatform facilitated abscopal radioimmune efficacy and notably suppressed lung tumor metastasis. In another report, Dong et al. prepared mesoporous organosilica nanoparticles (MONs) decorated with cancer cell membranes which contained X-ray and ROS dual-responsive diselenide bonds and were loaded with DOX for a synergistic ICD inducement [41]. Remarkably, the DOX-loaded MONs revealed impressive tumor growth inhibition and metastasis with negligible side effects.

**Figure 2:**
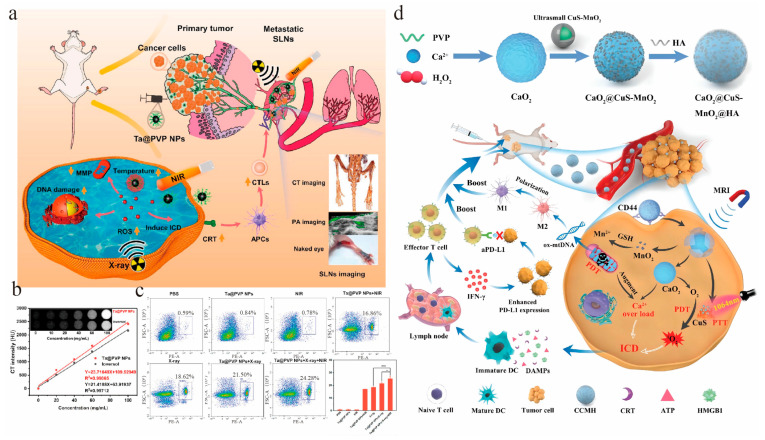
(**a**) Schematic illustration of polyvinylpyrrolidone (PVP)-coated Ta nanoparticles (Ta@PVP NPs) that enhanced therapeutic efficacy in primary tumor and metastatic sentinel lymph nodes (SLNs). (**b**) In vitro computed tomography (CT) intensity and images of Ioversol and Ta@PVP NPs. (**c**) Flow cytometry analysis of cytotoxic T lymphocytes (CTLs) in metastatic SLNs on the 20th day after treatments. ** *p* < 0.01; **** *p* < 0.0001. Reproduced with permission [42]. Copyright 2022, American Chemical Society. (**d**) Schematic of CaO_2_@CuS-MnO_2_@HA (CCMH) combined with NIR-II phototherapy for cancer immunotherapy. Reproduced with permission [51]. Copyright 2022, John Wiley and Sons.

To resolve the hypoxic-environment-mediated radiation resistance and high-radiation-dose-mediated safety issues, Chao Ji et al. designed polyvinylpyrrolidone (PVP)-coated tantalum (Ta) nanoparticles (Ta@PVP NPs) to improve blood flow via exploiting photothermal therapy (PTT) and enhance radiotherapy sensitivity via increasing intratumoral oxygen levels (Figure 2a) [42]. Ta@PVP NPs displayed a high antitumor efficacy in both in situ and metastatic breast tumors through radiosensitization. The real-time computed tomography (CT) imaging was monitored for the theranostic combination. As shown in Figure 2b, Ta@PVP NPs revealed a stronger CT signal than the clinical contrast agent Ioversol. When Ta@PVP NPs underwent X-Ray and NIR, the highest CTL percentage was detected, showing that RT and PTT enhanced antitumor immune responses. Liu et al. reported a poly(lactic-co-glycolic) acid (PLGA) nanoplatform with hydrophilic catalase (Cat) encapsulation in the core and the hydrophobic immune adjuvant imiquimod (R837) in the shell [43]. Owing to the ability of Cat to decompose hydrogen peroxide into oxygen, the PLGA nanoplatform alleviated hypoxia for potent radiotherapy-mediated ICD with tumor-associated antigen release. In the presence of adjuvant R837, the nanoplatform inhibited both primary as well as re-challenged tumor growth after combination with anticytotoxic T-lymphocyte associated protein 4 (CTLA4). Sun et al. used quantum dots with a near-infrared IIb window to deliver catalase, which also reversed hypoxic environments for enhanced radiotherapy and robust antitumor immune response [44]. However, the safety issues of the quantum dot nanoplatform, which may restrict its further application, should not be ignored [45].

## 3. Photodynamic Therapy

PDT relies on photosensitizers to generate ROS (e.g., hydroxyl radicals (•OH) and singlet oxygen (^1^O_2_)) under laser irradiation to directly kill tumor cells (Table 1) [46,47,48,49,50]. Studies have found that PDT could trigger ICD, consequently eliciting host immune responses [51,52,53,54,55].

The low oxygen content in the tumor microenvironment is adverse to PDT, resulting in reduced therapeutic effects. To address this issue, Huang et al. designed a nanoplatform CaO_2_@CuS-MnO_2_@HA (termed CCMH; CaO_2_: calcium peroxide; CuS: copper sulphide; MnO_2_: manganese dioxide; HA: hyaluronic acid) containing multiple ICD inducers (Figure 2d) [51]. In the acidic lysosome, CaO_2_ decomposed into Ca^2+^ and oxygen, resulting in calcium overload with mitochondrial damage and ICD inducement. CaO_2_ nanoparticles reacted with water to produce large amounts of oxygen that enhanced PDT efficacy. In addition, CuS nanoparticles produced ^1^O_2_ under 1064 nm irradiation, which also induced ICD. The authors claimed that the nanoplatform increased the expression of endoplasmic reticulum stress proteins BiP (Binding protein for immunoglobulins)/GRp78 (Glucose-regulated protein of 78 kDa), CHOP (C/EBP homologous protein)/DDIT3 (DNA Damage Inducible Transcript 3), and caspase-12 and promoted DAMP production. CaO_2_@CuS-MnO_2_@HA also caused TAM polarization via escaped oxidation-damaged mitochondrial DNA (ox-mtDNA) to activate the innate immune system. Although PDT triggers ROS generation, it also causes immunosuppressive cytokine release. In order to reverse the immunosuppressive effects, more and more researchers combine PDT with tumor microenvironment modulation for a better antitumor efficacy [56]. For example, Chen et al. synthesized a photodynamic immunomodulator, ICy-NLG, which was formed by covalently binding the near-infrared photosensitizer ICy-NH_2_ to the immune checkpoint inhibitor NLG919 via the disulfide bond [54]. After the disulfide bond cleavage by the intracellular GSH, the activity of the photosensitizer ICy was restored with tumor ICD, and the released NLG919 blocked indoleamine 2,3-dioxygenase (IDO-1) function with regulatory T cell inhibition. The synergistic use of photosensitizers and immune checkpoint inhibitors induced potent immune responses with a promising abscopal effect. Mai et al. constructed a carrier-free nanoparticle C9SN self-assembly from the photosensitizer Ce6 and the glutaminase inhibitor compound 968 (C968) [57]. Upon laser irradiation, C9SN produced a large number of ROS resulting in tumor ICD. Moreover, compound 968 inhibited glutamine metabolism, thereby inhibiting GSH production and disrupting REDOX homeostasis. Photodynamic therapy can also combine with chemoimmunotherapy for synergistic antitumor immunity. For example, Kim et al. fabricated a cathepsin B-responsive prodrug nanoplatform when DOX and the photosensitizer verteporfin (VPF) were conjugated via a cleavable linker Phe-Arg-ArgGly (FRRG) to form VPF-FRRG-DOX [58]. The authors found that only in the cancer cells with high expression of cathepsin-B, VPF and DOX were released for synergistic ICD with photo-/chemotherapy under laser irradiation. The prodrug platform displayed a strong antitumor activity as well as a durable immune memory response.

**Figure 3:**
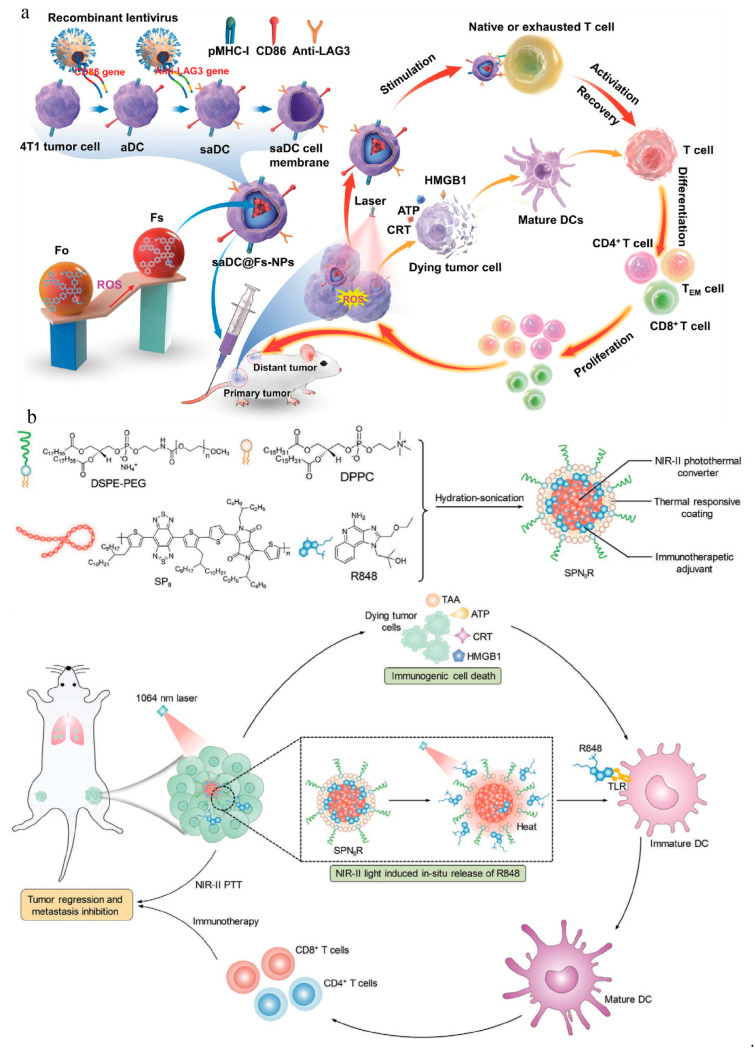
(**a**) Schematic diagram of the preparation process of saDC@Fs-NPs. saDC@Fs-NPs presented tumor antigens and reversed immunosuppression for PDT-enriched immunotherapy. saDC: super artificial dendritic cell. Reproduced with permission [59]. Copyright 2022, John Wiley and Sons. (**b**) Schematic illustration of semiconducting polymer nanoadjuvant (SPN_II_R) for NIR-II photothermal immunotherapy. Reproduced with permission [61]. Copyright 2020, John Wiley and Sons.

Tumor-specific immunity activation requires antigen-presenting cell (APC) maturation, tumor antigen presentation, and co-stimulatory signal activation. However, mature APCs have a short survival time in vivo, and the immunosuppressive microenvironment is another fact not to be ignored. To solve the above problems, Sun et al. designed a photodynamic biomimetic nanosystem when a super artificial dendritic cell (saDC) was constructed with CD86 and anti-LAG3 expression on a cell membrane to target 4T1 cells. In short, the polymer nanoskeleton loaded with an aggregation-induced emission (AIE) active photosensitizer, which was named Fs, was coated by an saDC membrane to form the biomimetic nanosystem saDC@Fs-NP (Figure 3a) [59]. In vivo results showed that, under laser irradiation, saDC@Fs-NPs reduced the proportion of bone-marrow-derived suppressor cells (MDSCs) with the immunosuppressive TME modulation, promoted the transformation of a “cold tumor” into a “hot one”, and thus enhanced the antitumor immune response. This nanosystem facilitated DC maturation, enhanced CTL infiltration, and reversed T cell exhaustion, which provides a new idea for the combination of PDT and immunotherapy. Chen et al. constructed size-transformable artificial antigen presenting cells (aAPCs) to combine photodynamic immunotherapy for an amplified antitumor immune response [5]. The aAPCs decorated with anti-CD28 and peptide major histocompatibility complex (MHC) displayed a size increment and prolonged retention time in tumor tissue when they encountered the preactivated T cells by PDT. This study opens a new approach to treat malignant tumors via aAPCs. Tang et al. utilized dendritic cell (DC) membrane loading with photosensitizer aggregation-induced emission to fabricate biomimetic nanosystems (DC@AIEdots) [60]. They found that DC@AIEdots, on the one hand, accumulated in the tumor tissue for PDT-mediated ICD, and also hitchhiked the endogenous T cell triggering for antitumor immunity.

**Table 1:** Summary of PDT-induced ICD to enhance cancer immune efficacy.

Formulation	Photosensitizer	Mechanism	NIR Light (nm)	Tumor Model	Ref.
Upconversion nanoparticle (UCNP)-based PDT system with an Fe-based CDT scaffold (UCS-PS-FeTA)	MC540Ce6	UCNPs loaded with dual photosensitizers were irradiated with near-infrared (NIR) light to generate ROS to induce PDT. FeTA reacts with H_2_O_2_ in the tumor microenvironment to induce CDT.	808	Hepa1-6, MC38	[62]
(Cytarabine, Ara-C)A-(Ce6)C/NPs	Ce6	The controllable triggering of GSDME-mediated pyroptosis via ROS accumulation leads to immunogenic cell death.	660	4T1, MDA-MB-231, MCF-7	[63]
Gold nanobipyramids and copper sulfifide in a core/shell architecture (AuNBP@CuS)	Semiconducto-rs/plasmonic metal	The accumulated electrons from plasmonic metal nanocrystals can be easily transferred to surface-adsorbed oxygen to form superoxide anions. The accumulated holes favor the formation of ^1^O_2_.	1064	EMT-6	[64]
Silk fibroin cRGDfk-Ce6 conjugate-based MnO_2_ nanocomposite (SRCM)	Ce6	The cRGDfk sequence enables SRCM to target solid tumors with high expression of αvβ3. SRCM is reduced by the acidic environment of lysosomes and glutathione in the cytoplasm. Ce6 is released, triggering PDT.	660	4T1	[65]
Au–Pd heterostructures (Au Pd HSs)	Plasmonic	Under laser irradiation, Au-Pd-HSs can generate a large number of hot electrons. These highly efficient hot electrons can immediately promote heat release and ROS production, including ^1^O_2_, superoxide radicals, and hydroxyl radicals.	808	4T1	[66]
M-LDH/ICG@Ca_3_(PO_4_)_2_, MICaP	ICG	ICG produces ^1^O_2_ to induce ICD.	808	4T1	[67]
Iron tungsten oxide (FeWOx)-based nanosheets with surface PEGylation (FWO-PEG NA)	FWO-PEG NA	The irradiation of FWO-PEG NA with 1060 nm light produced heat and ROS to achieve the CDT/PDT/PTT combination and to induce further ICD.	1060	4T1	[68]
Perylene monoamide-based ROS supergenerator (PMIC-NC)	PDIs	A hypoxia-enhanced burst of typeⅠROS is induced with the help of a proton transient, while the generation of typeⅠ/ⅡROS is triggered by electron or energy transfer under NIR light irradiation and triggers a strong ICD effect.	660	B16	[69]
RA-crosslinked supramolecular nanoassembly (CPR)	Ce6	CPR produces large amounts of ROS to damage tumor cells and induce ICD.	660	4T1	[70]

MC540: Merocyanine 540, Ce6: Chlorin-e6, PDIs: Perylenediimide derivatives.

## 4. Hyperthermia Therapy

Hyperthermic therapy (HT) refers to heating tissues to 39–45 °C, which induces endoplasmic reticulum stress through unfolded protein responses, upregulates heat shock proteins on a cell membrane surface, and releases TAAs, causing tumor ICD [71,72]. Photothermal therapy (PTT) is one type of HT, which means that a laser irradiates a tumor area, effectively converting light energy into heat energy through photothermal conversion agents [73]. Jingchao Li et al. used NIR-II semiconductor polymer nanoparticles (SPNs) and the toll-like receptor (TLR) agonist resiquimod (R848) to form a core, the surface of which was coated by a heat-responsive lipid shell to construct a multifunctional nanoplatform, SPN_II_R (Figure 3b) [61]. Under NIR II irradiation, SPN_II_R elicited PTT, which increased the local temperature of the tumor and induced ICD with DAMP and TAA release. In subcutaneous 4T1 tumor-bearing BALB/c mice, SPN_II_R-mediated photothermal immunotherapy not only enhanced the inhibitory effect on primary and distal tumor growth, but also significantly suppressed lung metastasis. In addition, the study not only achieved primary tumor ablation and ICD induction, but also achieved an in situ R848 release and remolded the tumor microenvironment.

**Figure 4:**
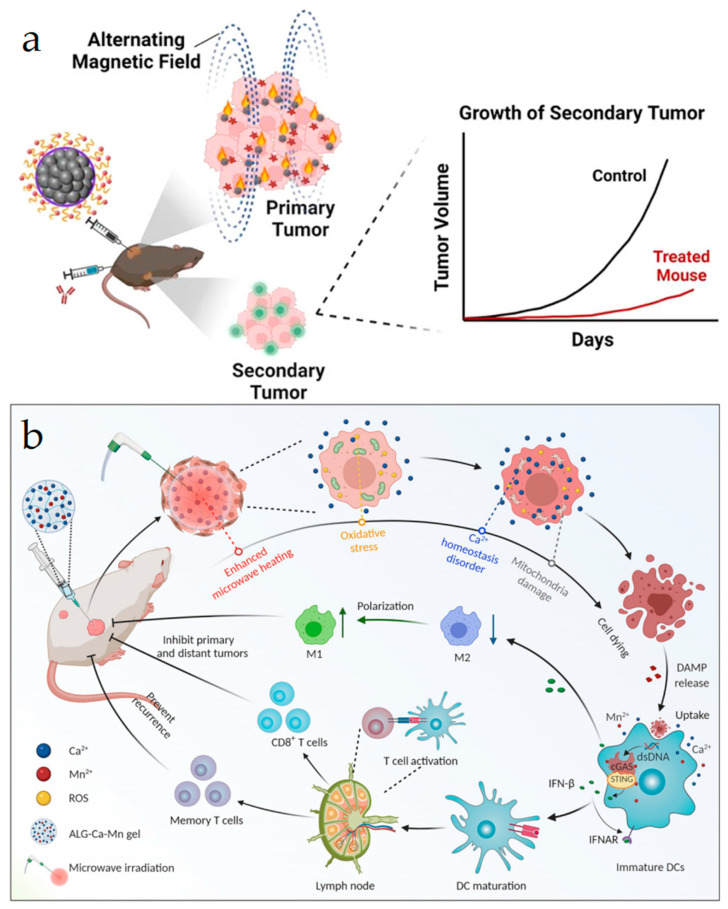
(**a**) ICD and dendritic cell maturation induced by IONC-AAPH-mediated magnetotherapy. IONC: iron oxide nanocrystals. AAPH: 2, 2′-azo compound (2-aminopropane) dihydrochloric acid. Reproduced with permission [81]. Copyright 2022, American Chemical Society. (**b**) A scheme illustrating the mechanism of metallo-alginate hydrogel in enhancing microwave ablation (MWA) treatment and boosting antitumor immunity. Reproduced with permission [82]. Copyright 2022, Amer Assoc Advancement Science.

Local thermal therapy (e.g., PTT and magnetic thermal therapy) has superior advantages, including negligible invasiveness and low side effects [71,74,75,76,77]. Multiple nanoformulations (e.g., inorganic nanoparticles, semiconductor polymeric nanoplatforms, and organic and inorganic hybrid nanomaterials) can be applied in thermal therapy with negligible toxicological issues which mainly achieve high tumor accumulation via the enhanced permeability and retention effect [61,78,79,80]. Li et al. developed a thermally responsive copolymer micelle PP_IR780_-ZMS-containing manganese zinc sulfide nanoparticle (ZMS) and a near-infrared dye IR780 [79]. Under NIR light irradiation, IR780 underwent photothermal conversion, thereby allowing PP_IR780_-ZMSs to release ZMSs for metal immunotherapy. The released ZMSs produced a large amount of Mn^2+^ with hydroxyl radical generation and ICD inducement. Simultaneously, Mn^2+^ activated the cGAS-STING pathway to produce type Ⅰ interferon and enhance tumor ICD. The results showed that PP_IR780_-ZMS elicited potent antitumor immune responses with lung tumor metastasis inhibition. Linlin Zhang et al. prepared a magnetic nanoplatform where the water-soluble 2, 2′-azo compound (2-aminopropane) dihydrochloric acid (AAPH) was encapsulated into magnetic iron oxide nanocrystals (IONCs) to form IONC-AAPH for photothermal and magnetic thermal therapy-mediated ICD [81]. In the presence of an alternating magnetic field, the heat induced by the azo compound facilitated carbon-centric radical generation (Figure 4a). Both magnetic heating and free radicals caused MC-38 tumor cell death, leading to DAMP release, DC maturation, CTL infiltration, and primary and distal tumor growth inhibition.

In addition, microwave ablation (MWA), which exploits polar molecules to cause strong oscillations under microwave irradiation, followed by heat generation with local thermal ablation of tumors, can also be used in tumor treatment. Yujie Zhu et al. designed a Ca^2+^-surplus alginate (ALG) saline gel which could increase heating efficiency and restrict the heating region under microwave irradiation [82]. As a highly effective microwave sensitizer, Ca^2+^ enhanced microwave heating in a concentration-dependent manner, leading to local thermal ablation and a large production of TAAs and DAMPs (Figure 4b). In addition, the synergistic effect of an extracellular high concentration of Ca^2+^ and hyperthermia induced ICD with intracellular Ca^2+^ homeostasis disruption. The results showed that ALG-Ca-assisted MWA treatment could effectively activate DCs in lymph nodes. Simultaneously, mice with ALG-Ca treatment revealed a high ratio of CD3^+^CD8^+^ to regulatory T cells (Tregs), indicating that the host antitumor immunity was activated. Du et al. built a thermal-responsive micellar nanoplatform that separately delivered adenosine receptor antagonist and doxorubicin (DOX) for both hyperthermia/chemotherapy-mediated ICD and immunosuppressive tumor microenvironment modulation under local microwave irradiation [83]. The E-selectin modification on the nanoplatform surface facilitated adherence to leukocytes for a higher tumor accumulation.

## 5. Chemodynamic Therapy

**Figure 5:**
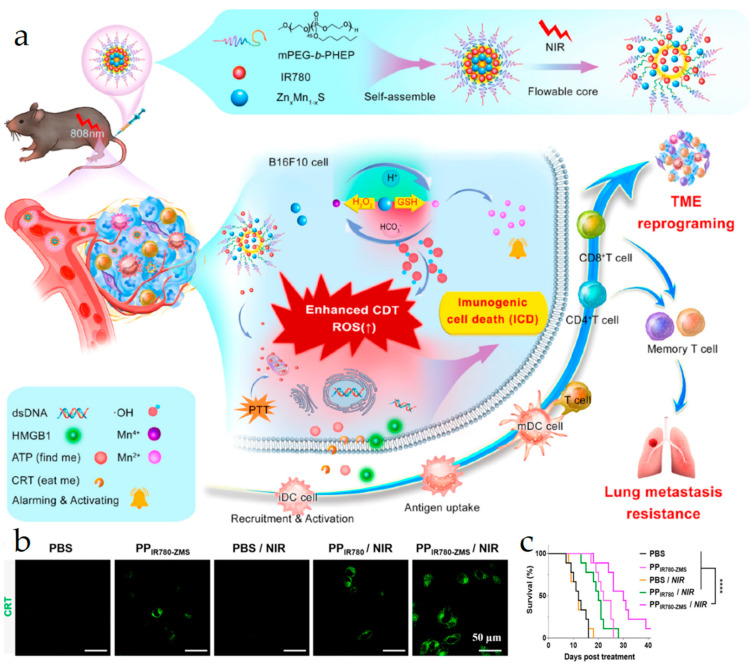
(**a**) Schematic illustration of self-assembly PP_IR780_-ZMSs releasing IR780 and ZMSs under NIR irradiation for tumor microenvironment education through enhanced CDT-associated immunogenic cell death. PP: polyethylene glycol (PEG)-poly (2-hexo-2-oxo-1,3, 2-dioxonane) copolymer (mPEG-b-PHEP); ZMSs: manganese zinc sulfide nanoparticles. (**b**) CLSM images and relative fluorescence intensity of CRT expression in B16F10 cells with the indicated treatments. (**c**) Survival curves of PBS, PP_IR780_-ZMS, PBS/NIR, PP_IR780_/NIR, and PP_IR780_-ZMS/NIR treated mice. **** *p* < 0.0001. Reproduced with permission [79]. Copyright 2022, American Chemical Society.

Chemodynamic therapy (CDT) refers to the use of tumor-endogenous hydrogen peroxide (H_2_O_2_) to generate toxic hydroxyl radicals through Fenton/Fenton-like reactions by metals (e.g., Fe^2+^, Mn^2+^, Mo^4+^, W^4+^, and Ti^3+^) [25,84,85,86,87,88,89,90,91,92,93,94]. CDT has attracted much attention due to its high specificity and selectivity for tumor tissues and negligible safety issues. Zhu Li et al. constructed a micellar nanoplatform, PP_IR780_-ZMS, which was self-assembled from polyethylene glycol (PEG)-poly (2-hexo-2-oxo-1,3, 2-dioxonane) copolymer (mPEG-*b*-PHEP) with IR780 and manganese zinc sulfide nanoparticle (ZMS) encapsulation in a thermosensitive core [79]. Under NIR light irradiation, IR780 underwent a photothermal transformation, facilitating a precise release of ZMSs from micelles. Mn^2+^ induced •OH generation to produce chemical kinetic efficacy, simultaneously eliciting ICD. In addition, the released IR780 accumulated in the mitochondria, leading to photothermal ablation, CDT efficacy enhancement, and DAMP secretion (Figure 5a). In vitro results showed an increased CRT exposure in B16F10 cells with PP_IR780-ZMS_/NIR treatment (Figure 5b). In vivo results showed that PP_IR780-ZMS_/NIR elicited a potent antitumor immune response in mice, significantly prolonged survival time (Figure 5c), and inhibited lung metastasis. Wang et al. reported gold nanoparticles which were decorated by a hollow mesoporous silica with manganese (Mn) doping and simultaneously with doxorubicin and aspirin (ASA) loading for ICD and dendritic cell maturation [95]. The gold nanoparticles had the activity of mimetic glucose oxidase converting glucose into hydrogen peroxide, which promoted Mn^2+^-mediated chemodynamic therapy with •OH generation. DOX and Mn^2+^-mediated tumor ICD combination with dendritic cell and T lymphocyte recruitment by ASA elicited a robust antitumor immunity.

By changing the 3D structures of constituent units in covalent organic frameworks (COFs), ROS are also able to be generated. Liang Zhang et al. used a COF platform to construct a three-dimensional structure of triphenylamine for ICD-mediated antitumor immunity activation [96]. The covalent linkage of COFs facilitated the generation and diffusion of ROS with endoplasmic reticulum stress for further tumor ICD with enhanced CD3^+^ CD8^+^ T cell infiltration. After combination with anti-CD47, both the primary and distal 4T1 tumor growth were notably inhibited.

**Figure 6:**
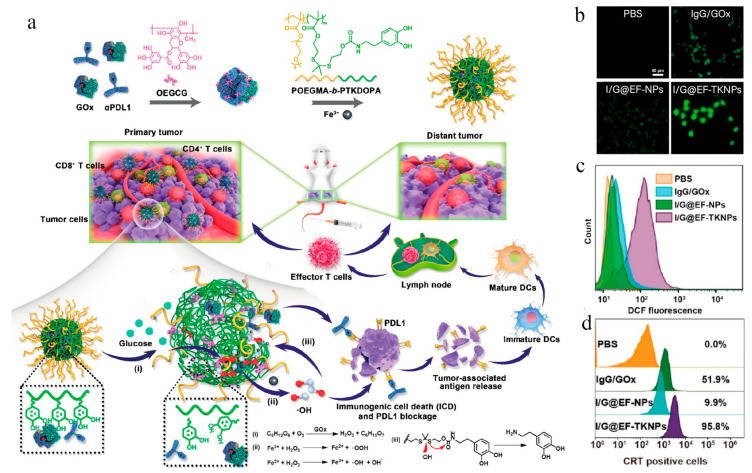
(**a**) Schematic illustration for the preparation of P/G@EF-TKNPs and the mechanism of in vivo combination therapy of CDT and immune checkpoint blockade (ICB). P/G: PDL1/GOx; EF: oligomerized (−)-epigallocatechin-3-O-gallate, Fe^3+^; TKNP: thioketal bond-linked nanoparticles. (**b**) Fluorescence images and (**c**) flow cytometry analysis of 4T1 cells after incubation for 24 h at the GOx-equivalent concentration of 100 mU mL^−1^ using the ROS fluorescence probe DCFH-DA. (**d**) Flow cytometric examination of CRT exposure on the surface of 4T1 tumor cells after various treatments [84]. Copyright 2022, American Chemical Society.

In addition, glucose depletion in the tumor microenvironment (TME) is also an effective tumor treatment approach after combination with CDT. Zhaoming Fu et al. fabricated a folate-modified metal–phenolic network nanosystem (F-MGC) with glucose oxidase (GOx) and chlorogenic acid (CHA) co-encapsulation [84,88]. Due to the catalytic activity of GOx and Fe^3+^, F-MGCs effectively consumed glucose in the TME and generated cytotoxic hydroxyl radicals for chemokinetic therapy accompanied by ICD. In vivo studies showed that CHA facilitated M2 phenotype TAM polarization into M1 type, remodeled the immunosuppressive TME, and activated the immune system. This nanoplatform exhibited an excellent biocompatibility and effectively inhibited tumor growth and metastasis. Li et al. developed ROS-responsive nanocomplexes that generated ROS via Fe^3+^-mediated Fenton reaction, delivering αPD-L1 and GOx (Figure 6a) [84]. At the tumor site, GOx that was released from the nanocomposites generated H_2_O_2_ through the oxidation of glucose, and Fe^3+^ induced •OH generation (Figure 6b,c) for CDT-mediated ICD (Figure 6d). The released αPD-L1 blocked PD-1/PDL1 interaction. Moreover, the released GOx increased the •OH production efficiency with the “cold” tumor microenvironment transformation into a “hot” one. Nanocomposites induced favorable systemic immune responses and effectively inhibited both primary and distant tumor growth in a bilateral 4T1 tumor model in mice.

## 6. Chemotherapy

Chemotherapeutic drugs (e.g., adriamycin, cisplatin, oxaliplatin, and doxorubicin) can cause tumor ICD when injected at a low dose (Table 2) [97,98,99]. However, in vivo low tumor accumulation as well as side effects are obstacles to the free drugs, which can be better resolved by nanotechnology [100].

Platinum (Pt) complexes are the most commonly used chemotherapeutic drugs approved in cancer treatment, while their clinical application is limited due to poor pharmacokinetics, severe side effects, and drug resistance. Jiajia Xiang et al. constructed tannic acid-Pt nanocomplex (PTI) nanoparticles, which produced tumor immunogenicity by inducing ICD, simultaneously improving antitumor efficacy after combination with immune checkpoint blockade (ICB) therapy [101]. Nanomedicine PTI was prepared in two steps. 1, 2-diaminocyclohexane-platinum (II) (DACHPt) was first complexed with natural polyphenols to form nanoparticles, which were then linked with iron (Ⅲ) by metal–catecholamine coordination bonds. PTI significantly promoted the expression of CRT on cell membranes, which revealed significantly higher exposure than that in other groups, proving its effective induction of ICD in vivo. The infiltration of CTL into the tumor was significantly increased after PTI treatment, which was 1.8-fold higher than that in the oxaliplatin group, and the secretion of IFN-γ and TNF-α was promoted. PTI significantly inhibited tumor growth, and the tumor inhibition rate was significantly higher than that in the control group (83%). In addition, PTI plus αPD-L1 significantly prolonged the survival rate of the mice, which was 100% at 44 days after inoculation, while that for PTI-alone treatment was only 37.5%.

**Figure 7:**
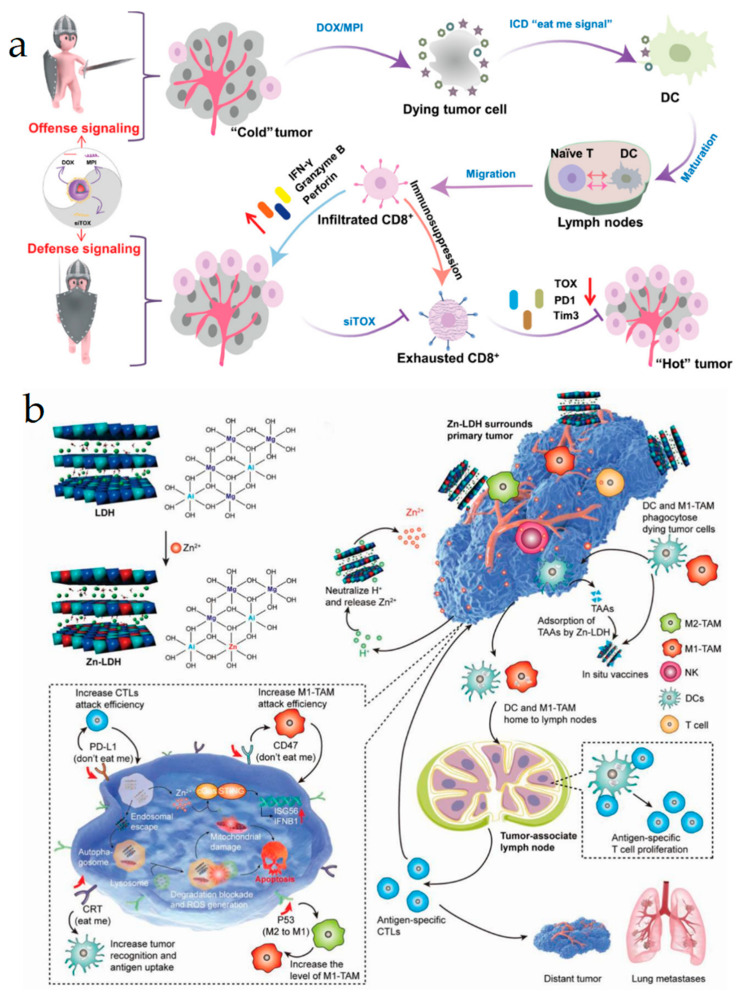
(**a**) Transition from “cold” to “hot” tumors depends on the manipulation of “offense and defense” signaling by FD/FM@siTOX NPs. FD (FDOX): Fluorinated prodrug doxorubicin; MPI: Peptide melittin; FM (FMPI): Fluorinated peptide melittin; TOX: Thymocyte selection-associated high-mobility group box protein. Reproduced with permission [102]. Copyright 2022, John Wiley and Sons. (**b**) Design and fabrication of an immunomodulating adjuvant that was doped with Zn^2+^ based on a double-layered hydroxide for robust and safe cancer metalloimmunotherapy. Reproduced with permission [105]. Copyright 2022, John Wiley and Sons.

Doxorubicin (DOX) is a classic ICD inducer, while the level of ICD induced by DOX alone is not enough to elicit a strong immune response. Pengkai Wu et al. developed a novel carrier-free nanoparticle that was able to deliver DOX, cytolytic peptide melittin (MPI), and an anti-TOX small-gap delivery RNA (siTOX) (Figure 7a) [102]. TOX is the abbreviation of thymocyte selection-associated high-mobility group box protein. pH-sensitive fluorinated prodrugs, FDOX (FD) and FMPI (FM), were synthesized to prepare FD/FM NPs through hydrophobic interactions and fluorine interactions. FD/FM@siTOX NPs were prepared by electrostatic interaction between FD/FM NPs and siTOX NPs. CD8^+^ T cell infiltration increased due to the combination of DOX and MPI with ICD enhancement. Mechanically, siTOX reduced TOX expression, which acted as a “defense” signal to reinvigorate CD8^+^ T cells. The author claimed that the constructed multifunctional nanosystem transformed a “cold” tumor into a “hot” one with effective antitumor immune responses during hepatoma treatment. Triple-negative breast cancer (TNBC) is prone to metastasis, possessing a cold immune microenvironment and poor prognosis [103,104]. Chemotherapy drugs are poorly targeted and have strong systemic toxicity. α5β1 integrin is over-expressed on breast cancer cells and plays an important role in tumor progression and metastasis. Xinyun Qiu et al. improved the targetability of nanocrystals by using a clinical Ac-PhScNK-NH_2_ (ATN) peptide to target α5β1 integrin [98]. α5β1 integrin-targeting micellar paclitaxel (ATN-MPTX) was constructed via using a reduction-sensitive biodegradable micelle as a carrier. ATN-MPTX induced DC maturation by PTX-mediated ICD. After combination with a STING agonist cyclic dinucleotide (CDN), ATN-MPTX significantly suppressed tumor growth and lung metastasis. Thus, compared with traditional chemotherapy, nanotechnology-assisted chemotherapeutics can achieve enhanced antitumor efficacy and decreased side effects via eliciting tumor ICD. They can also be combined with other approaches, including ICB and gene silencing, to achieve a better antitumor activity.

**Table 2:** Summary of nanoparticle-loaded chemotherapy-induced ICD to enhance cancer immune efficacy.

Formulation	Cargo	Treatment Modalities	Tumor Model	Ref.
Metallacycle-loaded nanoparticles (MNPs)	Tetraphenylethylene-based di-Pt(II) organometallic precursor (TPE-Pt) perylene bisimide fluorophore (PPy)	Chemotherapy, RT	A2780CIS-derived multicellular tumor spheroid (MCS)	[106]
Mannose-targeted RBCD vesicle-coated PLB/DIH co-loaded nanoformulation (Comb-NP)	PLB, DIH	Chemotherapy	Huh7 HCC	[107]
iRGD-modified BTZ-based nanomedicine (i-NPBTZ)	BTZ	Chemotherapy	4T1	[108]
DOX/POEG-bPCro micelles	DOX, Cro	Chemotherapy	4T1, LLC and HepG2	[109]
α5β1 integrin-targeted micellar paclitaxel (ATN-MPTX)	PTX	Chemotherapy, STING agonist	4T1	[98]
ACD (A: Au nanoclusters, C: copper ions, D: DOX)	DOX, Au nanozymes	Chemotherapy, CDT	4T1	[110]

PLB: Plumbagin, DIH: Dihydrotanshinone I, BTZ: Bortezomib, DOX: Doxorubicin, Cro: Crizotinib, PTX: Paclitaxel, CDT: chemodynamic therapy.

## 7. STING, Pyroptosis, Ferroptosis, and Autophagy-Induced ICD

In addition to the above strategies, it has been reported that ICD can also be induced by STING, pyroptosis, and ferroptosis [79,104,111,112,113].

The STING pathway is an important component of innate immunity and triggers productions of multiple immune stimulatory molecules, which can drive DC maturation, TAM polarization, and T cell priming, leading to immune-mediated tumor elimination. In addition, tumor ICD can also be induced by STING [114,115,116,117,118]. Lingxiao Zhang et al. constructed a Zn^2+^ layered zinc dihydroxide (Zn-LDH) nanoparticle, which consumed H^+^ with a large amount of Zn^2+^ release, accompanied by activation of the cGAS-STING pathway with tumor ICD [105]. Zn-LDH effectively destroyed lysosomes and blocked autophagy. In vivo results showed that Zn-LDH notably inhibited postoperative tumor growth and lung metastasis (Figure 7b).

In addition to the STING pathway, recent studies have found that pyroptosis can also induce ICD. Pyroptosis is an inflammatory form of programmed cell death characterized by cell swelling, giant bubble production, plasma membrane poring, chromatin fragmentation, and cytoplasmic proinflammatory cytokine extravasation. This process can enhance tumor ICD with TAA and DAMP release and promote tumor immunotherapy efficacy [63,119,120]. Lei Li et al. constructed carrier-free nanoplatform A-C/NPs with cytarabine (Ara-C) and chlorin e6 (Ce6) encapsulation [63]. A-C/NPs triggered GSDME-mediated pyroptosis in a controlled manner through ROS accumulation. The study showed that both Ce6 and A-C/NPs were able to trigger pyroptosis under near-infrared laser irradiation, and A-C/NPs had more swollen cells and more interleukin-1beta (IL-1β), IL-18, and NOD-like receptor family pyrin domain containing 3 (NLRP3) release than Ce6. A-C/NPs under laser irradiation effectively activated the systemic immune response in vivo. Seven days after surgical removal of the primary tumor, mice were subcutaneously inoculated with 4T1-Luc on the contralateral side, and A-C/NPs were found to effectively amplify an antigen-specific antitumor immune response and induce a long-lasting immune response. In another example, Li et al. developed an aggregation-induced emission photosensitive dimer that selectively targeted tumors, inducing ICD due to photodynamic and photothermal therapy [121]. Simultaneously, the ROS generation during PDT led to GSDME-mediated pyroptosis for an enhanced ICD with antitumor immunoefficacy.

Ferroptosis is a form of regulated cell death characterized by iron-dependent accumulation of lipid peroxides to lethal levels [122]. Ferroptosis has been found to effectively inhibit tumor growth accompanied by ICD [123,124,125,126]. Yaqian Du et al. reported a potent ROS production through a multifunctional nanoplatform, simultaneously leading to a depletion of glutathione peroxidase 4 (GPX4) and glutathione (GSH), which in total enhanced the occurrence of tumor ferroptosis and triggered tumor ICD [127]. The nanoplatform consisted of copper and iron silicate mesoporous hollow nanospheres, gold nanoparticles, and immune adjuvant R848. Fe^3+^ and Cu^2+^ could react with GSH to form glutathione disulfide (GSSG), leading to GSH depletion and GPX4 expression decrement. In addition, the formed Fe^2+^ could generate •OH via Fenton reaction, thus causing ferroptosis. It mainly activated host immune responses by causing ICD, increasing DC maturation and T-cell infiltration. The nanoplatform demonstrated a satisfactory efficacy in eliminating both primary and metastatic tumors. Yang et al. constructed a biomimetic magnetic nanoplatform with a glutamate-cystine antiporter system X_c_^−^ inhibitor sulfasalazine (SAS) encapsulation in the mesoporous Fe_3_O_4_ nanoparticle and platelet membrane decoration on the surface [128]. They found that the nanoplatform resulted in ferroptosis due to Fe_3_O_4_-mediated LPO accumulation and SAS-mediated system X_c_^−^ inhibition, which also generated immunogenicity and sensitized immune checkpoint blockade therapy.

Autophagy means that eukaryotic cells remove dysfunctional or unnecessary components to keep cellular homeostasis and manage metabolism [113,129,130]. It has a close relationship with tumor ICD when both autophagy inhibition and over-activation will lead to ICD. For example, Li et al. reported semiconducting polymer nanocomplexes with the autophagy inhibitor chloroquine (CQ) and IDO inhibitor NLG919 co-encapsulation [131]. CQ suppressed the degradation of intracellular damaged proteins or organelle resulting in PDT-mediated ICD enhancement, which then combined with NLG919-mediated immunometabolism suppression achieving a potent antitumor immunity. In another report, Wu et al. developed a nanomicelle dissolving microneedle (DMN) patch that could deliver the photosensitizer IR780 for PDT-mediated ICD and deliver CQ for autophagy inhibition-mediated damage-associated molecular pattern release [132]. The author also found that CQ facilitated TAM autophagy inhibition with M1 phenotype polarization for TME modulation. As for “over-activated” autophagy-mediated ICD, He et al. prepared a smart nanomicelle with oxaliplatin delivery resulting in ICD simultaneously accompanied by autophagy that cleaved the autophagy-responsive bond, leading to STF-62247 release [133]. As the autophagy inducer, STF-62247 caused autophagic death of tumor cells and facilitated tumor-associated antigen processing, thereby eliciting antitumor immune response.

Remarkably, in our previous report, we found that nanocarriers with tertiary amine and mercaptan structure also induced ICD selectively in B16F10 cells [127]. This finding opens a new avenue to exploiting nanomaterials for ICD-mediated cancer immunotherapy.

## 8. Conclusions

Immunotherapy has promising potential as a treatment strategy for many cancers. It mainly depends on activating the host immune system to attack pathological tissue. This review mainly summarized different nanotechnologies (e.g., RT, PDT, HT, CDT, and chemotherapy) mediating ICD for cancer immunotherapy. The detailed mechanisms and latest research advances for each approach were introduced. Simultaneously, the key issues, including hypoxia, tumor-associated macrophages, indoleamine 2,3-dioxygenase, as well as PD-1/PD-L1-mediated immunosuppression, were discussed in this review. The detailed strategies, including combination with TME modulation, cancer vaccination, ICB, and starvation therapy, were presented. In addition, the in vivo short survival time issue of DC was also mentioned, which was able to be resolved by artificial DC construction. We then introduced the relatively new aspects that STING, pyroptosis, ferroptosis, and nanocarriers could also lead to ICD, providing more strategies to potentiate ICD-mediated antitumor immune responses.

Even though nanomedicine-based tumor ICD has shown great promise in preclinical studies, there still exist some challenges. First, nanomedicines may have off-target effects. Second, some nanomaterials may be difficult to metabolize, resulting in long-term toxicity. Although biomimetic nanomaterials have unique advantages, they may have immunogenicity issues. Finally, exploring combinational therapies for synergistic effects is a likely necessity if dramatic improvements in patient survival rates are desired. Nanomedicines display huge superiority compared with free ICD inducers, including good in vivo stability, high tumor accumulation, controlled drug release, achievable combination therapy, and TME modulation. However, some nanomedicines may need complicated preparations and encounter problems in large-scale production. To achieve successful implementation in the clinic, the above issues should be resolved. In addition, the insurmountable gap between animals and humans may increase the clinical translation difficulty of nanomedicines when they display superior antitumor efficacy in cells/animals while being probably far less effective in humans. The simple design and good biodegradability of nanoparticles are essential for clinical translation.

## Data Availability

Not applicable.

## References

[1] Riley R.S., June C.H., Langer R., Mitchell M.J. (2019). Delivery technologies for cancer immunotherapy. Nat. Rev. Drug Discov..

[2] Topalian S.L., Weiner G.J., Pardoll D.M. (2011). Cancer immunotherapy comes of age. J. Clin. Oncol..

[3] DeLucia D.C., Lee J.K. (2022). Development of Cancer Immunotherapies. Cancer Treat. Res..

[4] Fan Y., Moon J.J. (2015). Nanoparticle Drug Delivery Systems Designed to Improve Cancer Vaccines and Immunotherapy. Vaccines.

[5] Yang W., Deng H., Zhu S., Lau J., Tian R., Wang S., Zhou Z., Yu G., Rao L., He L. (2020). Size-transformable antigen-presenting cell-mimicking nanovesicles potentiate effective cancer immunotherapy. Sci. Adv..

[6] Duan X., Chan C., Lin W. (2019). Nanoparticle-Mediated Immunogenic Cell Death Enables and Potentiates Cancer Immunotherapy. Angew. Chem. Int. Ed..

[7] Krysko D.V., Garg A.D., Kaczmarek A., Krysko O., Agostinis P., Vandenabeele P. (2012). Immunogenic cell death and DAMPs in cancer therapy. Nat. Rev. Cancer.

[8] Fu L., Ma X., Liu Y., Xu Z., Sun Z. (2022). Applying nanotechnology to boost cancer immunotherapy by promoting immunogenic cell death. Chin. Chem. Lett..

[9] Choi M., Shin J., Lee C.-E., Chung J.-Y., Kim M., Yan X., Yang W.-H., Cha J.-H. (2023). Immunogenic cell death in cancer immunotherapy. BMB Rep..

[10] Park S.J., Ye W., Xiao R., Silvin C., Padget M., Hodge J.W., Van Waes C., Schmitt N.C. (2019). Cisplatin and oxaliplatin induce similar immunogenic changes in preclinical models of head and neck cancer. Oral Oncol..

[11] Bai S., Zhang Y., Li D., Shi X., Lin G., Liu G. (2021). Gain an advantage from both sides: Smart size-shrinkable drug delivery nanosystems for high accumulation and deep penetration. Nano Today.

[12] Sahu T., Ratre Y.K., Chauhan S., Bhaskar L.V.K.S., Nair M.P., Verma H.K. (2021). Nanotechnology based drug delivery system: Current strategies and emerging therapeutic potential for medical science. J. Drug Deliv. Sci. Tec..

[13] Fang J., Islam W., Maeda H. (2020). Exploiting the dynamics of the EPR effect and strategies to improve the therapeutic effects of nanomedicines by using EPR effect enhancers. Adv. Drug Deliv. Rev..

[14] Shi Y., van der Meel R., Chen X., Lammers T. (2020). The EPR effect and beyond: Strategies to improve tumor targeting and cancer nanomedicine treatment efficacy. Theranostics.

[15] Ikeda-Imafuku M., Wang L.L., Rodrigues D., Shaha S., Zhao Z., Mitragotri S. (2022). Strategies to improve the EPR effect: A mechanistic perspective and clinical translation. J. Control. Release.

[16] Patra J.K., Das G., Fraceto L.F., Campos E.V.R., Rodriguez-Torres M.D.P., Acosta-Torres L.S., Diaz-Torres L.A., Grillo R., Swamy M.K., Sharma S. (2018). Nano based drug delivery systems: Recent developments and future prospects. J. Nanobiotechnol..

[17] Guo J., Zou Y., Huang L. (2023). Nano Delivery of Chemotherapeutic ICD Inducers for Tumor Immunotherapy. Small Methods.

[18] Dai H., Fan Q., Wang C. (2022). Recent applications of immunomodulatory biomaterials for disease immunotherapy. Exploration.

[19] Chen Q., Li C., Wang Q. (2023). Multifunctional Nano-Biomaterials for Cancer Therapy via Inducing Enhanced Immunogenic Cell Death. Small Methods.

[20] Zhao X., Yang K., Zhao R., Ji T., Wang X., Yang X., Zhang Y., Cheng K., Liu S., Hao J. (2016). Inducing enhanced immunogenic cell death with nanocarrier-based drug delivery systems for pancreatic cancer therapy. Biomaterials.

[21] Ding Y., Wang Y., Hu Q. (2022). Recent advances in overcoming barriers to cell-based delivery systems for cancer immunotherapy. Exploration.

[22] Jiang M., Zeng J., Zhao L., Zhang M., Ma J., Guan X., Zhang W. (2021). Chemotherapeutic drug-induced immunogenic cell death for nanomedicine-based cancer chemo-immunotherapy. Nanoscale.

[23] Shang T., Yu X., Han S., Yang B. (2020). Nanomedicine-based tumor photothermal therapy synergized immunotherapy. Biomater. Sci..

[24] Yang Y., Huang J., Liu M., Qiu Y., Chen Q., Zhao T., Xiao Z., Yang Y., Jiang Y., Huang Q. (2023). Emerging Sonodynamic Therapy-Based Nanomedicines for Cancer Immunotherapy. Sci. Adv..

[25] Tian H., Zhang M., Jin G., Jiang Y., Luan Y. (2021). Cu-MOF chemodynamic nanoplatform via modulating glutathione and H_2_O_2_ in tumor microenvironment for amplified cancer therapy. J. Colloid Interface Sci..

[26] Sun J., Huangfu Z., Yang J., Wang G., Hu K., Gao M., Zhong Z. (2022). Imaging-guided targeted radionuclide tumor therapy: From concept to clinical translation. Adv. Drug Deliv. Rev..

[27] Yu X., Ma H., Xu G., Liu Z. (2022). Radiotherapy assisted with biomaterials to trigger antitumor immunity. Chin. Chem. Lett..

[28] Huang Z., Yao D., Ye Q., Jiang H., Gu R., Ji C., Wu J., Hu Y., Yuan A. (2021). Zoledronic Acid-Gadolinium Coordination Polymer Nanorods for Improved Tumor Radioimmunotherapy by Synergetically Inducing Immunogenic Cell Death and Reprogramming the Immunosuppressive Microenvironment. ACS Nano.

[29] Pan P., Dong X., Chen Y., Ye J.J., Sun Y.X., Zhang X.Z. (2022). A heterogenic membrane-based biomimetic hybrid nanoplatform for combining radiotherapy and immunotherapy against breast cancer. Biomaterials.

[30] Zhu X., Wang X., Li B., Zhang Y., Chen Y., Zhang W., Wang Y., Zhai W., Liu Z., Liu S. (2022). A Three-In-One Assembled Nanoparticle Containing Peptide-Radio-Sensitizer Conjugate and TLR7/8 Agonist Can Initiate the Cancer-Immunity Cycle to Trigger Antitumor Immune Response. Small.

[31] Sun Q., Li J., Ding Z., Liu Z. (2023). Radiopharmaceuticals heat anti-tumor immunity. Theranostics.

[32] Li M., Wang Z., Liu X., Song N., Song Y., Shi X., Liu J., Liu J., Yu Z. (2021). Adaptable peptide-based therapeutics modulating tumor microenvironment for combinatorial radio-immunotherapy. J. Control. Release.

[33] Deng Z., Xi M., Zhang C., Wu X., Li Q., Wang C., Fang H., Sun G., Zhang Y., Yang G. (2023). Biomineralized MnO_2_ Nanoplatforms Mediated Delivery of Immune Checkpoint Inhibitors with STING Pathway Activation to Potentiate Cancer Radio-Immunotherapy. ACS Nano.

[34] Huang Z., Wang Y., Yao D., Wu J., Hu Y., Yuan A. (2021). Nanoscale coordination polymers induce immunogenic cell death by amplifying radiation therapy mediated oxidative stress. Nat. Commun..

[35] Li T., Gao M., Wu Z., Yang J., Mo B., Yu S., Gong X., Liu J., Wang W., Luo S. (2023). Tantalum-Zirconium Co-Doped Metal-Organic Frameworks Sequentially Sensitize Radio-Radiodynamic-Immunotherapy for Metastatic Osteosarcoma. Adv. Sci..

[36] Wang Y., Ding Y., Yao D., Dong H., Ji C., Wu J., Hu Y., Yuan A. (2021). Copper-Based Nanoscale Coordination Polymers Augmented Tumor Radioimmunotherapy for Immunogenic Cell Death Induction and T-Cell Infiltration. Small.

[37] Zheng D., Li Y., Song L., Xu T., Jiang X., Yin X., He Y., Xu J., Ma X., Chai L. (2022). Improvement of radiotherapy with an ozone-carried liposome nano-system for synergizing cancer immune checkpoint blockade. Nano Today.

[38] Zhu C., Guo X., Luo L., Wu Z., Luo Z., Jiang M., Zhang J., Qin B., Shi Y., Lou Y. (2019). Extremely Effective Chemoradiotherapy by Inducing Immunogenic Cell Death and Radio-Triggered Drug Release under Hypoxia Alleviation. ACS Appl. Mater. Interfaces.

[39] Choi B., Choi H., Yu B., Kim D.H. (2020). Synergistic Local Combination of Radiation and Anti-Programmed Death Ligand 1 Immunotherapy Using Radiation-Responsive Splintery Metallic Nanocarriers. ACS Nano.

[40] Qin X., Liu J., Xu Y., Li B., Cheng J., Wu X., Zhang J., Liu Z., Ning R., Li Y. (2020). Mesoporous Bi-Containing Radiosensitizer Loading with DOX to Repolarize Tumor-Associated Macrophages and Elicit Immunogenic Tumor Cell Death to Inhibit Tumor Progression. ACS Appl. Mater. Interfaces.

[41] Shao D., Zhang F., Chen F., Zheng X., Hu H., Yang C., Tu Z., Wang Z., Chang Z., Lu J. (2020). Biomimetic Diselenide-Bridged Mesoporous Organosilica Nanoparticles as an X-ray-Responsive Biodegradable Carrier for Chemo-Immunotherapy. Adv. Mater..

[42] Ji C., Zhao M., Wang C., Liu R., Zhu S., Dong X., Su C., Gu Z. (2022). Biocompatible Tantalum Nanoparticles as Radiosensitizers for Enhancing Therapy Efficacy in Primary Tumor and Metastatic Sentinel Lymph Nodes. ACS Nano.

[43] Chen Q., Chen J., Yang Z., Xu J., Xu L., Liang C., Han X., Liu Z. (2019). Nanoparticle-Enhanced Radiotherapy to Trigger Robust Cancer Immunotherapy. Adv. Mater..

[44] Li H., Wang M., Huang B., Zhu S.-W., Zhou J.-J., Chen D.-R., Cui R., Zhang M., Sun Z.-J. (2021). Theranostic near-infrared-IIb emitting nanoprobes for promoting immunogenic radiotherapy and abscopal effects against cancer metastasis. Nat. Commun..

[45] Kashyap B.K., Singh V.V., Solanki M.K., Kumar A., Ruokolainen J., Kesari K.K. (2023). Smart Nanomaterials in Cancer Theranostics: Challenges and Opportunities. ACS Omega.

[46] Son S., Kim J., Kim J., Kim B., Lee J., Kim Y., Li M., Kang H., Kim J.S. (2022). Cancer therapeutics based on diverse energy sources. Chem. Soc. Rev..

[47] Xu R., Chi W., Zhao Y., Tang Y., Jing X., Wang Z., Zhou Y., Shen Q., Zhang J., Yang Z. (2022). All-in-One Theranostic Platforms: Deep-Red AIE Nanocrystals to Target Dual-Organelles for Efficient Photodynamic Therapy. ACS Nano.

[48] Yang W., Zhu G., Wang S., Yu G., Yang Z., Lin L., Zhou Z., Liu Y., Dai Y., Zhang F. (2019). In Situ Dendritic Cell Vaccine for Effective Cancer Immunotherapy. ACS Nano.

[49] Shen L., Zhou T., Fan Y., Chang X., Wang Y., Sun J., Xing L., Jiang H. (2020). Recent progress in tumor photodynamic immunotherapy. Chin. Chem. Lett..

[50] Li M., Zhao M., Zhang Y., Ding M., Yu N., Peng S., Shi X., Li J. (2023). Second near-infrared light-activated semiconducting polymer nanomediators enable three-in-one tumor microenvironment modulation for combination immunotherapy. Nano Today.

[51] Huang C., Lin B., Chen C., Wang H., Lin X., Liu J., Ren Q., Tao J., Zhao P., Xu Y. (2022). Synergistic Reinforcing of Immunogenic Cell Death and Transforming Tumor-Associated Macrophages Via a Multifunctional Cascade Bioreactor for Optimizing Cancer Immunotherapy. Adv. Mater..

[52] Zhao L., Rao X., Huang C., Zheng R., Kong R., Chen Z., Yu X., Cheng H., Li S. (2022). Epigenetic reprogramming of carrier free photodynamic modulator to activate tumor immunotherapy by EZH2 inhibition. Biomaterials.

[53] Zheng R.R., Zhao L.P., Yang N., Chen Z.X., Kong R.J., Huang C.Y., Rao X.N., Chen X., Cheng H., Li S.Y. (2023). Cascade Immune Activation of Self-Delivery Biomedicine for Photodynamic Immunotherapy Against Metastatic Tumor. Small.

[54] Chen Y., Xiong T., Zhao X., Du J., Sun W., Fan J., Peng X. (2023). Tumor Cell-Responsive Photodynamic Immunoagent for Immunogenicity-Enhanced Orthotopic and Remote Tumor Therapy. Adv. Healthc. Mater..

[55] Liu J., Ai X., Cabral H., Liu J., Huang Y., Mi P. (2021). Tumor hypoxia-activated combinatorial nanomedicine triggers systemic antitumor immunity to effectively eradicate advanced breast cancer. Biomaterials.

[56] Jin F., Liu D., Xu X., Ji J., Du Y. (2021). Nanomaterials-Based Photodynamic Therapy with Combined Treatment Improves Antitumor Efficacy Through Boosting Immunogenic Cell Death. Int. J. Nanomed..

[57] Mai Z., Zhong J., Zhang J., Chen G., Tang Y., Ma W., Li G., Feng Z., Li F., Liang X.J. (2023). Carrier-Free Immunotherapeutic Nano-Booster with Dual Synergistic Effects Based on Glutaminase Inhibition Combined with Photodynamic Therapy. ACS Nano.

[58] Choi J., Shim M.K., Yang S., Hwang H.S., Cho H., Kim J., Yun W.S., Moon Y., Kim J., Yoon H.Y. (2021). Visible-Light-Triggered Prodrug Nanoparticles Combine Chemotherapy and Photodynamic Therapy to Potentiate Checkpoint Blockade Cancer Immunotherapy. ACS Nano.

[59] Sun Z., Liu J., Li Y., Lin X., Chu Y., Wang W., Huang S., Li W., Peng J., Liu C. (2022). Aggregation-Induced-Emission Photosensitizer-Loaded Nano-superartificial Dendritic Cells with Directly Presenting Tumor Antigens and Reversed Immunosuppression for Photodynamically Boosted Immunotherapy. Adv. Mater..

[60] Xu X., Deng G., Sun Z., Luo Y., Liu J., Yu X., Zhao Y., Gong P., Liu G., Zhang P. (2021). A Biomimetic Aggregation-Induced Emission Photosensitizer with Antigen-Presenting and Hitchhiking Function for Lipid Droplet Targeted Photodynamic Immunotherapy. Adv. Mater..

[61] Li J., Yu X., Jiang Y., He S., Zhang Y., Luo Y., Pu K. (2021). Second Near-Infrared Photothermal Semiconducting Polymer Nanoadjuvant for Enhanced Cancer Immunotherapy. Adv. Mater..

[62] Cen D., Zheng Q., Zheng B., Zhou R., Xiao X., Zhang T., Huang Z., Yan T., Yu J., Li X. (2023). A Near-Infrared Light-Responsive ROS Cascade Nanoplatform for Synergistic Therapy Potentiating Antitumor Immune Responses. Adv. Funct. Mater..

[63] Li L., Tian H., Zhang Z., Ding N., He K., Lu S., Liu R., Wu P., Wang Y., He B. (2023). Carrier-Free Nanoplatform via Evoking Pyroptosis and Immune Response against Breast Cancer. ACS Appl. Mater. Interfaces.

[64] Chen Y., Liu P., Zhou C., Zhang T., Zhou T., Men D., Jiang G., Hang L. (2023). Gold nanobipyramid@copper sulfide nanotheranostics for image-guided NIR-II photo/chemodynamic cancer therapy with enhanced immune response. Acta Biomater..

[65] He L., Wang J., Zhu P., Chen J., Zhao S., Liu X., Li Y., Guo X., Yan Z., Shen X. (2023). Intelligent manganese dioxide nanocomposites induce tumor immunogenic cell death and remould tumor microenvironment. Chem. Eng. J..

[66] Feng Y., Ning X., Wang J., Wen Z., Cao F., You Q., Zou J., Zhou X., Sun T., Cao J. (2023). Mace-Like Plasmonic Au-Pd Heterostructures Boost Near-Infrared Photoimmunotherapy. Sci. Adv..

[67] Zhu B., Qu F., Bi D., Geng R., Chen S., Zhu J. (2023). Monolayer LDH Nanosheets with Ultrahigh ICG Loading for Phototherapy and Ca^2+^-Induced Mitochondrial Membrane Potential Damage to Co-Enhance Cancer Immunotherapy. ACS Appl. Mater. Interfaces.

[68] Xiang Q., Yang C., Luo Y., Liu F., Zheng J., Liu W., Ran H., Sun Y., Ren J., Wang Z. (2022). Near-Infrared II Nanoadjuvant-Mediated Chemodynamic, Photodynamic, and Photothermal Therapy Combines Immunogenic Cell Death with PD-L1 Blockade to Enhance Antitumor Immunity. Small.

[69] Lou X., Wang H., Liu Y., Huang Y., Liu Z., Zhang W., Wang T. (2023). Perylene-Based Reactive Oxygen Species Supergenerator for Immunogenic Photochemotherapy against Hypoxic Tumors. Angew. Chem. Int. Ed..

[70] Zhou Y., Zhang Y., Jiang C., Chen Y., Tong F., Yang X., Wang Y., Xia X., Gao H. (2023). Rosmarinic Acid-Crosslinked Supramolecular Nanoassembly with Self-Regulated Photodynamic and Anti-Metastasis Properties for Synergistic Photoimmunotherapy. Small.

[71] Ge J., Yang N., Yang Y., Yu H., Yang X., Wang Y., Wang T., Cheng S., Wang Y., Han Z. (2023). The combination of eddy thermal effect of biodegradable magnesium with immune checkpoint blockade shows enhanced efficacy against osteosarcoma. Bioact. Mater..

[72] Jiang H., Fu H., Guo Y., Hu P., Shi J. (2022). Evoking tumor associated macrophages by mitochondria-targeted magnetothermal immunogenic cell death for cancer immunotherapy. Biomaterials.

[73] Chang M., Hou Z., Wang M., Li C., Lin J. (2021). Recent Advances in Hyperthermia Therapy-Based Synergistic Immunotherapy. Adv. Mater..

[74] Li Q., Fan H., Xu Y., Liu M., Liu J., Xu L., Zou M., Cheng Q., Zhang Y., Liang T. (2023). NIR-responsive hollow germanium nanospheres mediate photothermal/photodynamic therapy and restrain immunosuppression to cooperatively eradicate primary and metastatic tumors. Chem. Eng. J..

[75] Zhao Q., Liang G., Guo B., Wang W., Yang C., Chen D., Yang F., Xiao H., Xing N. (2023). Polyphotosensitizer-Based Nanoparticles with Michael Addition Acceptors Inhibiting GST Activity and Cisplatin Deactivation for Enhanced Chemotherapy and Photodynamic Immunotherapy. Sci. Adv..

[76] Sun Q., Yang Z., Lin M., Peng Y., Wang R., Du Y., Zhou Y., Li J., Qi X. (2021). Phototherapy and anti-GITR antibody-based therapy synergistically reinvigorate immunogenic cell death and reject established cancers. Biomaterials.

[77] Liu X., Zheng C., Kong Y., Wang H., Wang L. (2022). An in situ nanoparticle recombinant strategy for the enhancement of photothermal therapy. Chin. Chem. Lett..

[78] Li X., Lovell J.F., Yoon J., Chen X. (2020). Clinical development and potential of photothermal and photodynamic therapies for cancer. Nat. Rev. Clin. Oncol..

[79] Li Z., Chu Z., Yang J., Qian H., Xu J., Chen B., Tian T., Chen H., Xu Y., Wang F. (2022). Immunogenic Cell Death Augmented by Manganese Zinc Sulfide Nanoparticles for Metastatic Melanoma Immunotherapy. ACS Nano.

[80] Li J., Lu W., Yang Y., Xiang R., Ling Y., Yu C., Zhou Y. (2022). Hybrid Nanomaterials for Cancer Immunotherapy. Adv. Sci..

[81] Zhang L., Zhang Q., Hinojosa D.T., Jiang K., Pham Q.K., Xiao Z., Colvin V.L., Bao G. (2022). Multifunctional Magnetic Nanoclusters Can Induce Immunogenic Cell Death and Suppress Tumor Recurrence and Metastasis. ACS Nano.

[82] Zhu Y., Yang Z., Pan Z., Hao Y., Wang C., Dong Z., Li Q., Han Y., Tian L., Feng L. (2022). Metallo-alginate hydrogel can potentiate microwave tumor ablation for synergistic cancer treatment. Sci. Adv..

[83] Qi J., Jin F., You Y., Du Y., Liu D., Xu X., Wang J., Zhu L., Chen M., Shu G. (2021). Synergistic effect of tumor chemo-immunotherapy induced by leukocyte-hitchhiking thermal-sensitive micelles. Nat. Commun..

[84] Li X., Zhou Q., Japir A., Dutta D., Lu N., Ge Z. (2022). Protein-Delivering Nanocomplexes with Fenton Reaction-Triggered Cargo Release to Boost Cancer Immunotherapy. ACS Nano.

[85] Ding F., Li F., Tang D., Wang B., Liu J., Mao X., Yin J., Xiao H., Wang J., Liu Z. (2022). Restoration of the Immunogenicity of Tumor Cells for Enhanced Cancer Therapy via Nanoparticle-Mediated Copper Chaperone Inhibition. Angew. Chem. Int. Ed..

[86] Chen M., Yang J., Zhou L., Hu X., Wang C., Chai K., Li R., Feng L., Sun Y., Dong C. (2022). Dual-Responsive and ROS-Augmented Nanoplatform for Chemo/Photodynamic/Chemodynamic Combination Therapy of Triple Negative Breast Cancer. ACS Appl. Mater. Interfaces.

[87] Feng X., Lin T., Chen D., Li Z., Yang Q., Tian H., Xiao Y., Lin M., Liang M., Guo W. (2023). Mitochondria-associated ER stress evokes immunogenic cell death through the ROS-PERK-eIF2α pathway under PTT/CDT combined therapy. Acta Biomater..

[88] Fu Z., Zhang Y., Chen X., Wang N., Ma R., Luo X., Pan X., Yang Y., Xue W. (2022). A Versatile Nanoplatform Based on Metal-Phenolic Networks Inhibiting Tumor Growth and Metastasis by Combined Starvation/Chemodynamic/Immunotherapy. Adv. Funct. Mater..

[89] Huang Y., Wu S., Zhang L., Deng Q., Ren J., Qu X. (2022). A Metabolic Multistage Glutathione Depletion Used for Tumor-Specific Chemodynamic Therapy. ACS Nano.

[90] Kuai X., Zhu Y., Yuan Z., Wang S., Lin L., Ye X., Lu Y., Luo Y., Pang Z., Geng D. (2022). Perfluorooctyl bromide nanoemulsions holding MnO_2_ nanoparticles with dual-modality imaging and glutathione depletion enhanced HIFU-eliciting tumor immunogenic cell death. Acta Pharm. Sin. B.

[91] Liu Y., Zhai S., Jiang X., Liu Y., Wang K., Wang C., Zhang M., Liu X., Bu W. (2021). Intracellular Mutual Promotion of Redox Homeostasis Regulation and Iron Metabolism Disruption for Enduring Chemodynamic Therapy. Adv. Funct. Mater..

[92] Yan J., Yu H., Tang X., Li F., Li Z., Liang Y., He B., Wang X., Sun Y. (2023). Highly triple-effective synergy based on tetrahedral DNA nanostructure-induced tumor vaccines for cancer therapy. Mater. Design.

[93] Zhou Y., Fan S., Feng L., Huang X., Chen X. (2021). Manipulating Intratumoral Fenton Chemistry for Enhanced Chemodynamic and Chemodynamic-Synergized Multimodal Therapy. Adv. Mater..

[94] Bai Y., Pan Y., An N., Zhang H., Wang C., Tian W., Huang T. (2023). Host-guest interactions based supramolecular complexes self-assemblies for amplified chemodynamic therapy with H_2_O_2_ elevation and GSH consumption properties. Chin. Chem. Lett..

[95] Sun K., Hu J., Meng X., Lei Y., Zhang X., Lu Z., Zhang L., Wang Z. (2021). Reinforcing the Induction of Immunogenic Cell Death Via Artificial Engineered Cascade Bioreactor-Enhanced Chemo-Immunotherapy for Optimizing Cancer Immunotherapy. Small.

[96] Zhang L., Yang L.L., Wan S.C., Yang Q.C., Xiao Y., Deng H., Sun Z.J. (2021). Three-Dimensional Covalent Organic Frameworks with Cross-Linked Pores for Efficient Cancer Immunotherapy. Nano Lett..

[97] Guo B., Qu Y., Sun Y., Zhao S., Yuan J., Zhang P., Zhong Z., Meng F. (2023). Co-delivery of gemcitabine and paclitaxel plus NanoCpG empowers chemoimmunotherapy of postoperative “cold” triple-negative breast cancer. Bioact. Mater..

[98] Qiu X., Qu Y., Guo B., Zheng H., Meng F., Zhong Z. (2022). Micellar paclitaxel boosts ICD and chemo-immunotherapy of metastatic triple negative breast cancer. J. Control. Release.

[99] Yang J., Ma S., Xu R., Wei Y., Zhang J., Zuo T., Wang Z., Deng H., Yang N., Shen Q. (2021). Smart biomimetic metal organic frameworks based on ROS-ferroptosis-glycolysis regulation for enhanced tumor chemo-immunotherapy. J. Control. Release.

[100] Zhang X., Wang S., Cheng G., Yu P., Chang J., Chen X. (2021). Cascade Drug-Release Strategy for Enhanced Anticancer Therapy. Matter.

[101] Xiang J., Zhang Y., Liu X., Zhou Q., Piao Y., Shao S., Tang J., Zhou Z., Xie T., Shen Y. (2022). Natural Polyphenols-Platinum Nanocomplexes Stimulate Immune System for Combination Cancer Therapy. Nano Lett..

[102] Wu P., Zhang H., Sun M., Mao S., He Q., Shi Y., Deng Y., Dong Z., Xu Q., Zhao C. (2022). Manipulating Offense and Defense Signaling to Fight Cold Tumors with Carrier-Free Nanoassembly of Fluorinated Prodrug and siRNA. Adv. Mater..

[103] Li C.W., Lim S.O., Hsu J.L., Hung M.C. (2017). Rational combination of immunotherapy for triple negative breast cancer treatment. Chin. Clin. Oncol..

[104] Zhang Q., Shi D., Guo M., Zhao H., Zhao Y., Yang X. (2023). Radiofrequency-Activated Pyroptosis of Bi-Valent Gold Nanocluster for Cancer Immunotherapy. ACS Nano.

[105] Zhang L., Zhao J., Hu X., Wang C., Jia Y., Zhu C., Xie S., Lee J., Li F., Ling D. (2022). A Peritumorally Injected Immunomodulating Adjuvant Elicits Robust and Safe Metalloimmunotherapy against Solid Tumors. Adv. Mater..

[106] Ding Y., Tong Z., Jin L., Ye B., Zhou J., Sun Z., Yang H., Hong L., Huang F., Wang W. (2022). An NIR Discrete Metallacycle Constructed from Perylene Bisimide and Tetraphenylethylene Fluorophores for Imaging-Guided Cancer Radio-Chemotherapy. Adv. Mater..

[107] Han S., Bi S., Guo T., Sun D., Zou Y., Wang L., Song L., Chu D., Liao A., Song X. (2022). Nano co-delivery of Plumbagin and Dihydrotanshinone I reverses immunosuppressive TME of liver cancer. J. Control. Release.

[108] Jiang W., Zhou H., Wang Q., Chen Z., Dong W., Guo Z., Li Y., Zhao W., Zhan M., Wang Y. (2021). High drug loading and pH-responsive nanomedicines driven by dynamic boronate covalent chemistry for potent cancer immunotherapy. Nano Res..

[109] Liang Q., Lan Y., Li Y., Cao Y., Li J., Liu Y. (2022). Crizotinib prodrug micelles co-delivered doxorubicin for synergistic immunogenic cell death induction on breast cancer chemo-immunotherapy. Eur. J. Pharm. Biopharm..

[110] Zhao D.H., Li C.Q., Hou X.L., Xie X.T., Zhang B., Wu G.Y., Jin F., Zhao Y.D., Liu B. (2021). Tumor Microenvironment-Activated Theranostics Nanozymes for Fluorescence Imaging and Enhanced Chemo-Chemodynamic Therapy of Tumors. ACS Appl. Mater. Interfaces.

[111] Wang Z., Chen J., Hu J., Zhang H., Xu F., He W., Wang X., Li M., Lu W., Zeng G. (2019). cGAS/STING axis mediates a topoisomerase II inhibitor-induced tumor immunogenicity. J. Clin. Investig..

[112] Chen C., Wang Z., Jia S., Zhang Y., Ji S., Zhao Z., Kwok R.T.K., Lam J.W.Y., Ding D., Shi Y. (2022). Evoking Highly Immunogenic Ferroptosis Aided by Intramolecular Motion-Induced Photo-Hyperthermia for Cancer Therapy. Adv. Sci..

[113] Gao W., Wang X., Zhou Y., Wang X., Yu Y. (2022). Autophagy, ferroptosis, pyroptosis, and necroptosis in tumor immunotherapy. Signal Transduct. Target. Ther..

[114] Chattopadhyay S., Liu Y.H., Fang Z.S., Lin C.L., Lin J.C., Yao B.Y., Hu C.J. (2020). Synthetic Immunogenic Cell Death Mediated by Intracellular Delivery of STING Agonist Nanoshells Enhances Anticancer Chemo-immunotherapy. Nano Lett..

[115] Zhang R., Kang R., Tang D. (2021). The STING1 network regulates autophagy and cell death. Signal Transduct. Target..

[116] Li C., Zhang Y., Wan Y., Wang J., Lin J., Li Z., Huang P. (2021). STING-activating drug delivery systems: Design strategies and biomedical applications. Chin. Chem. Lett..

[117] Garland K.M., Sheehy T.L., Wilson J.T. (2022). Chemical and Biomolecular Strategies for STING Pathway Activation in Cancer Immunotherapy. Chem. Rev..

[118] Samson N., Ablasser A. (2022). The cGAS–STING pathway and cancer. Nat. Cancer.

[119] Ma X., Su W., Ye M., Gao Y., Qiu W., Liang M., Xue P., Kang Y., Sun Z.-J., Xu Z. (2023). Endogenous/exogenous stimulies inspired polyprodrug nano-inducer switches pyroptosis path for promoting antitumor immunity. Nano Today.

[120] Wang H., Gao Z., Jiao D., Zhang Y., Zhang J., Wang T., Huang Y., Zheng D., Hou J., Ding D. (2023). A Microenvironment Dual-Responsive Nano-Drug Equipped with PD-L1 Blocking Peptide Triggers Immunogenic Pyroptosis for Prostate Cancer Self-Synergistic Immunotherapy. Adv. Funct. Mater..

[121] Tang Y., Bisoyi H.K., Chen X.M., Liu Z., Chen X., Zhang S., Li Q. (2023). Pyroptosis-Mediated Synergistic Photodynamic and Photothermal Immunotherapy Enabled by Tumor Membrane-Targeted Photosensitive Dimer. Adv. Mater..

[122] Stockwell B.R., Friedmann Angeli J.P., Bayir H., Bush A.I., Conrad M., Dixon S.J., Fulda S., Gascon S., Hatzios S.K., Kagan V.E. (2017). Ferroptosis: A Regulated Cell Death Nexus Linking Metabolism, Redox Biology, and Disease. Cell.

[123] Yang C., Wang M., Chang M., Yuan M., Zhang W., Tan J., Ding B., Ma P., Lin J. (2023). Heterostructural Nanoadjuvant CuSe/CoSe_2_ for Potentiating Ferroptosis and Photoimmunotherapy through Intratumoral Blocked Lactate Efflux. J. Am. Chem. Soc..

[124] Luo L., Wang H., Tian W., Li X., Zhu Z., Huang R., Luo H. (2021). Targeting ferroptosis-based cancer therapy using nanomaterials: Strategies and applications. Theranostics.

[125] Yu B., Choi B., Li W., Kim D.H. (2020). Magnetic field boosted ferroptosis-like cell death and responsive MRI using hybrid vesicles for cancer immunotherapy. Nat. Commun..

[126] Zafar H., Raza F., Ma S., Wei Y., Zhang J., Shen Q. (2021). Recent progress on nanomedicine-induced ferroptosis for cancer therapy. Biomater. Sci..

[127] Du Y., Zhang R., Yang J., Liu S., Zhou J., Zhao R., He F., Zhang Y., Yang P., Lin J. (2022). A “Closed-Loop” Therapeutic Strategy Based on Mutually Reinforced Ferroptosis and Immunotherapy. Adv. Funct. Mater..

[128] Jiang Q., Wang K., Zhang X., Ouyang B., Liu H., Pang Z., Yang W. (2020). Platelet Membrane-Camouflaged Magnetic Nanoparticles for Ferroptosis-Enhanced Cancer Immunotherapy. Small.

[129] Ishimwe N., Zhang W., Qian J., Zhang Y., Wen L. (2020). Autophagy regulation as a promising approach for improving cancer immunotherapy. Cancer Lett..

[130] Xia H., Green D.R., Zou W. (2021). Autophagy in tumour immunity and therapy. Nat. Rev. Cancer.

[131] Yu N., Ding M., Wang F., Zhou J., Shi X., Cai R., Li J. (2022). Near-infrared photoactivatable semiconducting polymer nanocomplexes with bispecific metabolism interventions for enhanced cancer immunotherapy. Nano Today.

[132] Chen M., Yang D., Sun Y., Liu T., Wang W., Fu J., Wang Q., Bai X., Quan G., Pan X. (2021). In Situ Self-Assembly Nanomicelle Microneedles for Enhanced Photoimmunotherapy via Autophagy Regulation Strategy. ACS Nano.

[133] Wang X., Li M., Ren K., Xia C., Li J., Yu Q., Qiu Y., Lu Z., Long Y., Zhang Z. (2020). On-Demand Autophagy Cascade Amplification Nanoparticles Precisely Enhanced Oxaliplatin-Induced Cancer Immunotherapy. Adv. Mater..

